# Self-Supervised Robust Feature Matching Pipeline for Teach and Repeat Navigation

**DOI:** 10.3390/s22082836

**Published:** 2022-04-07

**Authors:** Tomáš Rouček, Arash Sadeghi Amjadi, Zdeněk Rozsypálek, George Broughton, Jan Blaha, Keerthy Kusumam, Tomáš Krajník

**Affiliations:** 1 Artificial Intelligence Center, Faculty of Electrical Engineering, Czech Technical University in Prague, 166 27 Prague 6, Czech Republic; amjadara@fel.cvut.cz (A.S.A.); rozsyzde@fel.cvut.cz (Z.R.); george.broughton@fel.cvut.cz (G.B.); jan.blaha@fel.cvut.cz (J.B.); krajnt1@fel.cvut.cz (T.K.); 2 Department of Computer Science, University of Nottingham, Jubilee Campus, 7301 Wollaton Rd, Lenton, Nottingham NG8 1BB, UK; keerthy.kusumam2@nottingham.ac.uk

**Keywords:** visual teach and repeat navigation, long-term autonomy, self-supervised machine learning, computer vision, mobile robot, artificial neural network, deep learning

## Abstract

The performance of deep neural networks and the low costs of computational hardware has made computer vision a popular choice in many robotic systems. An attractive feature of deep-learned methods is their ability to cope with appearance changes caused by day–night cycles and seasonal variations. However, deep learning of neural networks typically relies on large numbers of hand-annotated images, which requires significant effort for data collection and annotation. We present a method that allows autonomous, self-supervised training of a neural network in visual teach-and-repeat (VT&R) tasks, where a mobile robot has to traverse a previously taught path repeatedly. Our method is based on a fusion of two image registration schemes: one based on a Siamese neural network and another on point-feature matching. As the robot traverses the taught paths, it uses the results of feature-based matching to train the neural network, which, in turn, provides coarse registration estimates to the feature matcher. We show that as the neural network gets trained, the accuracy and robustness of the navigation increases, making the robot capable of dealing with significant changes in the environment. This method can significantly reduce the data annotation efforts when designing new robotic systems or introducing robots into new environments. Moreover, the method provides annotated datasets that can be deployed in other navigation systems. To promote the reproducibility of the research presented herein, we provide our datasets, codes and trained models online.

## 1. Introduction

Cameras are one of the most efficient and widely used sensors, especially in robotics. To mention some prominent advantages of cameras, they are affordable, small, and lightweight, without any interference with each other. Besides, they provide high-resolution real-time data that do not impose computational problems thanks to powerful computational hardware getting cheaper over time. Considering these advantages, onboard cameras are regularly used in mobile robots to gather information about the surrounding environment [1,2], and are particularly attractive for smaller robots with limited payload [3,4,5].

In the context of mobile robot navigation, cameras are often used to segment out traversable areas, such as roads or pathways [6], or they are used to create environment maps that are used for localisation [7,8,9] or directly for navigation [10,11]. Many of these methods rely on the use of algorithms that extract local image features, e.g., SIFT or ORB [12,13], which are then matched to features collected during mapping and the resulting correspondences are used to establish the robot position or direction. The authors of [14] compared the performance of several feature extractors and concluded that for visual simultaneous localisation and mapping (SLAM), the SIFT [12] provide superior performance. However, the authors of [15,16,17] pointed out that the maps created by visual SLAM can become obsolete over time due to seasonal variations and other natural changes.

The issue of long-term stability of vision-based maps for mobile robot navigation was also investigated in the context of visual teach-and-repeat (VTR), where the main task of the robot is to repeat a previously taught trajectory. Some of the efforts aimed at the use of multiple maps [18,19] or image features tailored for long-term VTR [20,21,22]. While these approaches have demonstrated a certain degree of progress towards the long-term operation of mobile robots, the advent of deep learning (DL) brought the possibility to train image processing methods robust to seasonal changes [23,24,25,26,27]. While the methods based on deep-learned neural networks outperform those based on engineered features, their training typically requires large volumes of images. These training sets have to contain subsets of images that capture the same scenes that appear differently over time. This makes training data collection and annotation a tedious and time-consuming task, which is a major bottleneck in the deployment of these systems.

**Figure 1:**
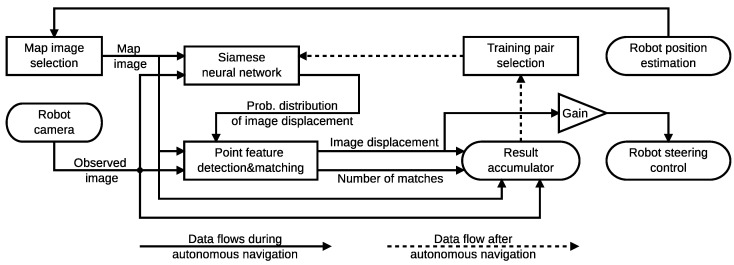
Core part of the proposed ‘visual teach, repeat and learn’ (VTRL) navigation pipeline. The siamese neural network provides priors to the feature matching, which, in turn, provides registration results to train the neural network. This results in gradual improvement of VTR robustness.

Inspired by the fact that engineered features can achieve vision-based teach-and-repeat navigation in the short-term and deep-learned neural networks can do the same in the long-term, we propose a VTR pipeline that combines them in a fusion-learning scheme. The proposed pipeline, shown in Figure 1, utilises an artificial neural network (hereafter simply called neural network) [28] to provide priors for a feature matching algorithm [20], that determines the heading of the robot relative to the taught path. Selected image pairs along with the heading they were used to establish are subsequently used as training samples for the neural network. Initially, the neural network is untrained, so the first autonomous traversal of the taught path is based purely on feature matching. After the first autonomous run, the neural network is trained and starts to provide priors to the feature matcher, increasing its robustness. Each subsequent run provides more images for the training of the neural network, gradually improving the efficiency of the navigation pipeline. Since the training samples constitute image pairs, their number increases quadratically with each autonomous traversal, which ensures that enough samples can be obtained to train the network properly. Any noise present in the labels during the training of the neural network should have limited impact provided the false positive and false negative rate is not too significant, due to the fact that the network is trying to learn the simplest possible way to interpret the data. Therefore small numbers of incorrect labels will not provide a consistent learning target, while all the correctly annotated data will. We hypothesise that the proposed scheme can train the neural network without any human supervision and outperform the hand-engineered feature matching in term of robustness to significant seasonal changes.

In this paper, we contribute to state of the art by investigating the impact of the aforementioned self-supervised training in the context of visual teach-and-repeat navigation. We show that the robustness of VTLR gradually increases despite the seasonal changes, while the error of the classic VTR gradually increases. Moreover, we deploy the self-trained method in a different environment and show that it outperforms classic VTR as well. Finally, we compare the performance of the self-trained VTLR to VTLR, which uses ground truth data for training.

The rest of the paper is organized as follows. In Section 2, we provide a brief state of the art and review other VTR navigation systems and the image processing techniques they use. Then, in Section 3, we describe our system architecture and the individual methods, i.e., the navigation module, neural network description, training sample filtering and self-supervised learning. In Section 4, we show what datasets have been used throughout this paper and provide an overview of their main properties. Later, in Section 5, we investigate the robustness of the proposed “Visual Teach, Repeat and Learn” (VTRL) navigation as a robot traverses the same path that undergoes significant appearance changes due to seasonal factors. The results of the investigation are discussed in Section 6. Finally, we provide a conclusion of the work and possible future research directions.

## 2. Related Work

The use of computer vision has a long tradition in robotics, and robot vision methods are becoming sufficiently mature enough to be deployed in real-world systems. This applies especially to the field of visual SLAM, where a robot with a single camera can precisely estimate its position while creating a map of its operational environment [7,8], and visual navigation, where image analysis methods are used in the control loop to guide mobile robots [1,10,11]. Nevertheless, as the robustness of these methods allowed increased operation time of the robots, new challenges related to varying illumination and environment changes started to arise. Researchers using standard feature-based localisation and mapping methods started to point out that the long-term robustness of feature-based localisation is not satisfactory [15].

This challenge attracted researchers to propose solutions that would allow robots to operate over extended periods of time, where environment changes are inevitable. In [29], the wavelength distribution of the sun was employed to calculate images with invariance to illumination changes. Deploying this method in robot vision improved the performance of robots in outdoor environments with varying illumination and shadows [30,31,32,33]. However, the method decreases the performance of visual processing in situations where the Sun is not a prominent source of light, such as indoors and at night [17]. Moreover, the method is not designed to deal with other variances such as environmental changes.

To boost the robots’ ability of long-term operation, several authors proposed various feature management schemes aimed at a gradual update of the maps to keep up with the environment changes. Some of the works aim to assess the temporal stability of individual features in sparse visual maps in order to update features that are going to be stable in the long-term while removing those that are no longer useful [34,35,36,37,38]. The approach is comprehensibly summarised in [39], which evaluates several methods for managing the features over multiple mapping sessions performed over the period of several months.

Other approaches aimed to tackle the problem by using several maps gathered during multi-session mapping runs. In particular, Churchill and Newman [40] proposed the concept of ‘experiences’ which represent different appearances of the same locations under different conditions. This approach was extended by taking into account temporal properties of the environmental conditions, which lead to efficient management of the experiences [40,41]. The experience-based approach was integrated into visual teach and repeat systems [18], which were reported to traverse over 1000 km over the period of several days [19,42].

Another stream of research proposed not only to remember (and forget) the past experiences but to analyse them in order to forecast the future environment’s appearance. Neubert et al. proposed a method that can learn appearance changes based on a cross-seasonal dataset and use the learned model to predict the environment appearance [21,43]. Lowry et al. [44] used linear regression techniques in image space to forecast the appearance of different locations in different conditions. Other works proposed to use Fourier analysis or dynamic system theory to learn the temporal patterns of feature visibility and use that to construct maps relevant for a particular time [45,46]. The map forecasting schemes were integrated into a visual teach-and-repeat system as well and compared to approaches that aim at map adaptation [47]. A gradual introduction of deep learning in the field of robot vision brought even more efficient methods of forecasting the environment appearance through the use of generative adversarial networks (GANS) [48,49,50].

Along with the aforementioned works, several teams contributed by collecting multi-session mapping datasets that captured the appearance and structural changes over long periods of time. Datasets like Nordland [21], KAIST day–night [51], KITTY multi-session [52], North Campus [53], Oxford car [54] or EU long-term [55] allowed not only to test the performance of the vision-based localisation and navigation methods, but also to train data-hungry machine learning algorithms.

Some of these machine learning methods aimed to train classic feature descriptors in order to increase their robustness to seasonal changes. For example, the pixel brightness comparison sequence of the Binary Robust Independent Elementary Features (BRIEF) [56] was trained on the aforementioned datasets, and the result, called GRIEF [20] was shown to perform similarly as another deep learned feature [57]. The performance of GRIEF was further improved by using different comparison sequences depending on the location [22]. Other authors trained and tested convolutional [24,26,58], siamese [28] or generative adversarial neural networks [49,50] in the context of long-term SLAM or visual teach-and-repeat navigation [26,27] in changing environments.

Overall, there was a significant work on long-term visual localisation and navigation in changing environments and it’s beyond the scope if this paper to systematise all the approaches. A number of survey papers provides a good insight into the problem. In particular, ref. [59] survey the field of SLAM from the perspective of robustness and reliability, ref. [17] provides an overview of vision-based localisation techniques, ref. [60] aims at methods enabling long-term autonomy and a recent survey in [61] aims at visual SLAM in particular.

### Point Feature Detectors and Descriptors

Several of the aforementioned pipelines employ heavily engineered or hand-crafted features which gained huge popularity in robotic systems, due to their efficient memory usage and performance. The design of the image features aims at achieving invariance towards natural changes occurring in images such as illumination, occlusions, geometric transformations etc. and involves two stages, namely, detection and description [12,13,15]. During the detection stage, the detector identifies key regions of interest such as corners, blobs, edges within an image, known as the keypoints. The description algorithm then specifies a method to summarize the region surrounding the keypoints and provides a descriptor vector, which captures mainly local textures or gradients. The features are robust and provide invariance to view-point, illumination, clutter and natural changes.

The best known method for feature extraction is the Scale Invariant Feature Transform (SIFT) [12] which utilizes a Difference of Gaussians detector to identify scale-invariant keypoints and the descriptor uses gradient orientation histograms to form a 128 dimensional vector. The SIFT was widely used for its stability, however it runs slower compared to other features and until recently was protected by a patent.

Another well known combination of detector and descriptor is the Speeded Up Robust Features (SURF) which uses a Hessian keypoint detector and an optimised description from SIFT [15]. The SURF is significantly faster than SIFT but is still under patent protection.

A truly open-source combination is the Oriented FAST and Rotated BRIEF (ORB) [13] which uses modified oFAST detector that is based on pixel intensities arranged in a circular pattern and rBRIEF descriptor that utilises binary strings for the encoding of the keypoint and a set map for comparison while the features are being matched. The ORB [13] is faster, more robust and rotation invariant and was built for object recognition and visual SLAM techniques. However ORB or BRIEF internally use comparison patterns which are random or chosen manually. A Generated BRIEF (GRIEF) [62] attempts to generate the comparison pattern by initially training using a genetic algorithm and applied to Teach and Repeat Navigation, yielding better results.

## 3. Method

Our goal is to design a method that would allow visual teach-and-repeat navigation in changing environments over long periods of time. The navigation pipeline is based on a teach-and-repeat method that uses the visual data to correct the heading of a robot to keep it on the intended path [10,11,63,64,65]. We assume that the robot was taught by driving the robot along the intended path by a human and that during the drive, the robot was able to collect images and index them by the traveled distance, e.g., by odometry or dead-reckoning. The theory behind the teach-and-repeat methods points out (for polygonal [11,66] and smooth [24,65] paths) that teach-and-repeat methods do not require full six degree-of-freedom global localisation. Rather, they can simply use the currently perceived and previously stored images to determine the robot heading relative to the path it was taught. Correct estimation of the heading ensures convergence of the robot trajectory to the intended path [11,65]. This heading is estimated through the use of feature- or neural-network-based image registration, which can be further simplified as the heading correction is proportional to the horizontal displacement of the mapped and perceived images.

Our augmented feature detection and matching pipeline consist of two main parts, feature matcher (FM) and neural network (NN), see Figure 1. Given the perceived image I and previously recorded image $I^{\prime }$, retrieved from the map the neural network [28] first estimates their coarse registration and provides this information to the feature matcher. The feature matcher uses this coarse estimate to establish the correspondences of point-based features and uses traditional feature matching [20] along with a histogram voting scheme to determine the horizontal displacement of the images. The calculated displacement is then used to steer the robot. Moreover, the displacement, along with the number of features and the corresponding image pair is also stored in the result accumulator. After the robot completes the autonomous run, image pairs with high number of feature-based correspondences are retrieved from the accumulator and used to train the network. After the training, the neural network should be able to provide better priors to improve the quality of the feature matching.

### 3.1. Robot Navigation

Our pipeline is integrated the BearNav [65] visual teach-and-repeat navigation, which uses the ROS (Robot Operating System) framework. The Bearnav navigation system combines the odometric and camera data obtained during the teaching phase to repeat the taught path autonomously. During the teaching phase, the robot stores the forward and steering speeds according to the travelled distance and captures images of the environment in regular intervals using its forward-facing camera. After the teaching phase is completed, the pipeline simply replays the forward velocity, while correcting the steering (i.e., angular velocity) based on the horizontal displacement of the currently visible image with the corresponding map one. While simple, this type of navigation has been mathematically proven to eliminate injected position errors and to converge to the taught path even in presence of significant disturbances caused by wheel slippage [11,65]. While the system does not require traditional camera calibration, it needs to set a gain constant that translates the map-to-current image displacement to the steering velocity of the robot. This constant depends on the mechanical properties of the robot and resolution and field of view of the camera used. Overall, the robot steering ω is calculated as $\omega =\omega_{r}+\alpha \;d$, where $\omega_{r}$[rad] is retrieved from the odometric information stored in the map, *d* is the displacement of the images in pixels and α is the steering gain, see Figure 1. This type of navigation does not bypass obstacles in the taught path and stationary obstacles have to be handled by a complementary method as described in [11]. Dynamic obstacles typically intersect the robot trajectory only temporarily, which can be solved by slowing down or pausing the navigation.

### 3.2. Neural Network

**Figure 2:**
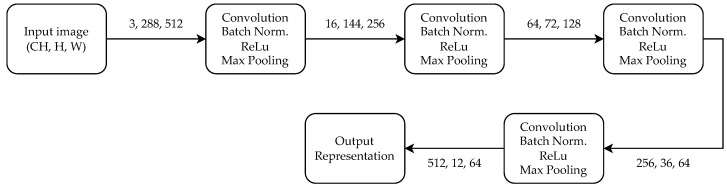
Schematics of the backbone’s architecture.

The role of the neural network is to estimate the likelihood of horizontal displacements between two images—one from the map and one currently perceived by the robot. For that reason, the neural network is built so that it’s output is a histogram of likelihood values corresponding to possible displacements. The NN model used is a fully convolutional Siamese neural network with a backbone similar to AlexNet [67] inspired by our previous works on neural networks for teach-and-repeat navigation [24,28]. The network is relatively shallow so it can run on a mobile robot with limited computation capabilities. In order to make the final results position-dependent, no fully connected layers were used in the output layer. The backbone is shown in Figure 2.

The input of the network are two images I and $I^{\prime }$. Both images are passed through the backbone of the network. The backbone’s output is a neural representation of individual images, which should be robust to changes in the scene appearance after proper training. The backbone’s architecture is designed so that the width of the obtained representation is eight times smaller than the input image. This choice is a compromise between robustness and accuracy. Given the pair of images, one representation is used as a convolutional kernel, which is applied to the representation of the second image. We are adding roll padding in the horizontal axis of the second image’s representation so that the convolution outputs the cross-correlation of the representations with different horizontal displacements. The value of cross-correlation over all possible displacements is normalized and gives a probability of the displacement. The internal NN accepts images with a width of 512 pixels and due to the padding used, it creates 63 likelihood values each corresponding to a possible shift of an image by 8 pixels. If a larger image is processed by our pipeline, it is first downsampled to 512 pixel width and then the final output corresponds to $\frac{8}{512}\cdot w$, where *w* is the input image width. The final output of NN for each image pair therefore is a probability distribution over 63 possible pixel shifts between the two images. This is then used as a prior by our modified feature matching scheme.

### 3.3. Feature Matching

In a standard FM module, keypoints in images I and $I^{\prime }$ are detected and vectors describing their pixel neighbourhood are calculated. Then, the distance between these vectors is used to establish the correspondences between the features in I and $I^{\prime }$. To remove the outlying correspondences, the coordinates of the matched features are checked for consistency; this is typically performed by employing known principles in epipolar geometry. For our pipeline, we used the FAST [68] detector and GRIEF [20] descriptor as this combination performs favourably for long-term visual teach and repeat [20]. In our experiments, we also tested the ORB [13] and SIFT [12] features, however, the achieved results were inferior to the ones obtained by FAST/GRIEF combination.

In a standard matching method, the extracted features form two sets F and $F^{\prime }$ extracted from the images I and $I^{\prime }$, respectively. These can be matched either using a ‘symmetric match’, which considers features $f_{0}\in F$ and $f_{0}^{\prime }\in F^{\prime }$ a pair if a given feature $f_{0}$ is the nearest neighbour of $f_{0}^{\prime }$ in the set $F^{\prime }$ and vice versa. Another possibility is the ‘ratio test’, which searches the descriptor space for two nearest neighbours $f_{0}^{\prime },f_{1}^{\prime }\in F^{\prime }$ to a given feature $f_{0}\in F$. A match is formed if

(1) $$\left|\right|f_{0}-f_{0}^{\prime }\left||\;\;<\;\;r\;|\right|f_{0}-f_{1}^{\prime }\left|\right|,$$

where *r* is typically chosen around 0.8 as stated in the original paper [12]. In our pipeline, we use the ‘ratio test’ matching with the *r* coefficient set to 0.95.

However, in contrast to the standard feature matching, our module exploits the probabilistic distribution over possible displacements provided by the neural network. This is in the form of a probability mass function with values $p_{d}$, for every d∈{−31…31} denoting individual possible shifts of the neural representation. For a particular value of *d*, the value $p_{d}$ then gives the probability of the image displacement being in the interval of (8d−4,8d+4] pixels. The aforementioned values hold for images with 512 pixel width. Images of different size would result in scaling these values accordingly as explained in Section 3.2. To take the values of $p_{d}$ into account, we first find the maximal probability $p_{d}$, designate it as $p_{d}^{m}$ and add the interval (8d−4,8d+4] it to an (empty) set of intervals P. After that, we select all the values $p_{d}$ that are higher than $0.7\;p_{d}^{m}$ and add their respective intervals to the set P. The set ${⋃}_{J\in P}J$ then contains the permitted values of horizontal displacement between the potentially matched features. Then, we start to iterate over the features in the set F and for each feature $f_{i}$ with an image coordinate $\left(u_{i},v_{i}\right)$, we search the set $F^{\prime }$ for features with coordinates $\left(u_{j}^{\prime },v_{j}^{\prime }\right)$ such as the difference of their horizontal coordinates $d_{ij}=\left(u_{i}-u_{j}^{\prime }\right)$ lies inside of some interval in P. The features satisfying the aforementioned conditions are then matched to $f_{i}^{\prime }$ using the ratio test matching and the resulting matches $\left(f_{i},f_{j}^{\prime }\right)$ are added to a set M.

This filtering scheme has two effects: First, it prevents from matching features with positions that are inconsistent with the prior proposed by the neural network, reducing the number of false correspondences. Second, it improves the number of matches, because the ratio match is performed for lower number of features and thus, the chance of satisfying the ratio test in Equation (1) is higher.

The image coordinates of the matches $\left(f_{i},f_{j}^{\prime }\right)$, i.e., $\left(u_{i},v_{i}\right),\left(u_{j}^{\prime },v_{j}^{\prime }\right)$ are then used to calculate a set of horizontal displacements $D=\{u_{i}-u_{j}^{\prime }\;|\;\left(f_{i},f_{j}^{\prime }\right)\;\in \;M\}$. Finally, the modus *d* of the set D is determined using the histogram voting scheme. The value of *d* is then considered as a horizontal displacement between I and $I^{\prime }$, and the number of matches m=|M| represents the quality of the estimation of *d*. The tuple $\left(I,I^{\prime },d,m\right)$ is then stored in the result accumulator C for postprocessing and the value of *d* is passed to the robot steering controller.

### 3.4. Training Pair Selector and Result Accumulator

Once the robot completes the path, the training pair selector starts to process the information stored in the result accumulator C to retrieve the training pairs for the Neural Network. In our method, we search all the results $(I_{l},I_{l}^{\prime },d_{l},m_{l})\in C$ to find the one with the highest value of matches $m_{l}$, i.e., $m_{max}$. Then, it populates the training set T, with the all the samples $\left(I_{t},I_{t}^{\prime },d_{t},m_{t}\right)$ that satisfy the condition $m_{t}>0.25\;m_{max}$. While the reduced set T is typically half the size of C, it contains a much smaller ratio of incorrectly matched pairs that could hamper the learning process.

The aforementioned filtering could be complemented by manual examination and rejection of incorrect training pairs. However as will be shown in Section 6, the results achieved are not worse compared to supervised training which requires human annotators.

### 3.5. Neural Network Training

The training scheme of the neural network is a bit different to the evaluation scheme already described in this section. Instead of taking whole images $I_{t}$ and $I_{t}^{\prime }$ and adding padding to one of them, we take one whole image and only a small random cut out (56 pixels wide) from the second image. In this setup, it is unnecessary to add significant padding to one of the embeddings during the training.

**Figure 3:**
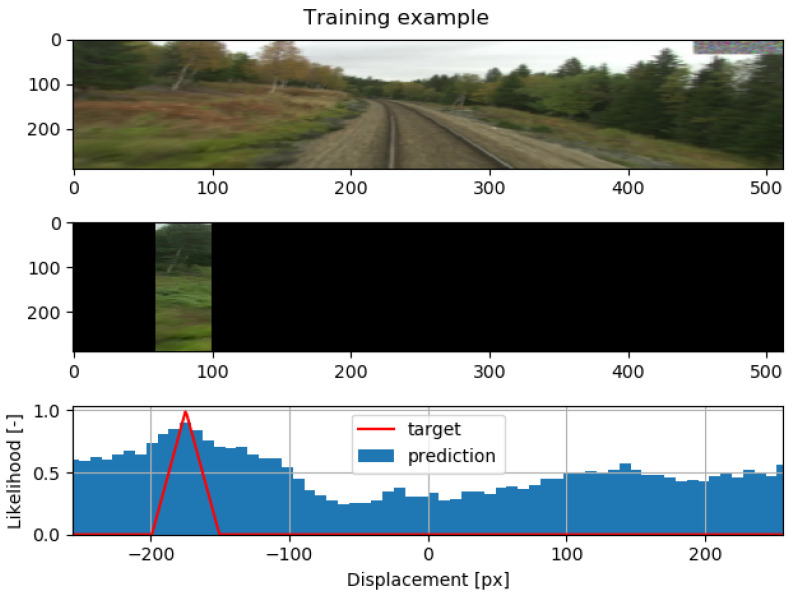
In this figure, we show the training procedure. We obtained a pair of corresponding images with seasonal variations from the training set. The second image is cropped, and the target is created accordingly. The target is set to one at its location on the first image and then decreases towards the cutout edges.

The target for the training pair is constructed to reflect the position of the cutout in the original image and the displacement $d_{t}$ between the images obtained by the feature matcher. The visualization of one training sample and its target is shown in Figure 3. Similarly to [28], the loss function *L* is calculated as binary cross-entropy between the prediction and the target as

(2) $$L=\frac{\sum_{n=0}^{N}y_{n}\log x_{n}+\left(1-y_{n}\right)\log\left(1-x_{n}\right)}{N}\;,$$

where the $x_{n}$ is the *n*th element of the output of the NN bounded by a sigmoid to range of [0,1], $y_{n}$ is the *n*th element of the target position displacement and *N* is the total number of all possible horizontal displacement between the image and the cutout.

## 4. Datasets

To evaluate the proposed pipeline, we followed the methodology proposed in [20,24,28] and simulated a teach-and-repeat scenario using three multi-session long-term datasets. The results of the image displacement calculations were then compared to the ground truth, and the errors were used to assess the quality of the visual teach-and-repeat navigation.

### 4.1. Nordland

To simulate lifelong learning, the teaching was done in several batches on Nordland dataset [69]. This dataset was recorded in 2012 on the Norway rail network that spans 729-km and contains images captured during four seasons. The gathering was performed using a camera mounted on a front of a train that has driven the same path on a regular basis. From the original dataset, which contains about 3 million images, we have selected images that are taken further apart from each other, which resulted in approximately 55,000 images divided into four groups, each captured during a different drive. Each drive occurred during a different season, and the associated dataset contains 14,123 images with 512 × 288 pixel resolution.

**Figure 4:**
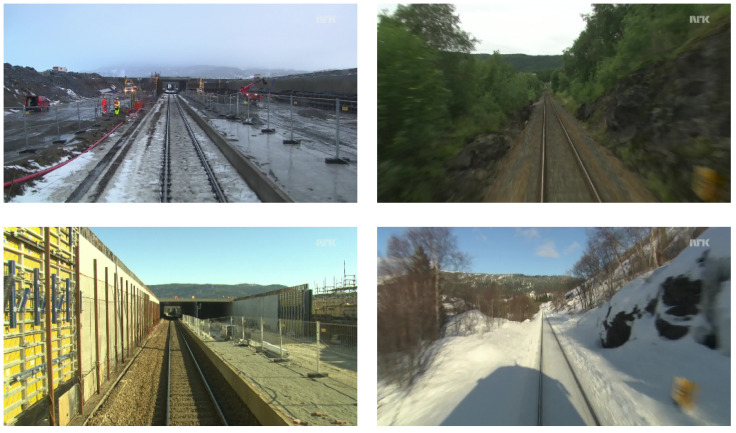
View of Nordland dataset on two different places. Changes in seasons from summer to winter (**right**) and tunnel being built (**left**).

The Nordland dataset includes the same track most of the time, and it captures heavy seasonal changes such as snow and fog and change in foliage as well as human-made changes such as newly constructed tunnels Figure 4. However, the images of the dataset contain a small watermark in the top right corner and a railway in the middle bottom of the image. These were removed to prevent over-fitting of the network. In particular, the watermark was blurred using Gaussian noise, and the railway was cropped out completely. To train the neural network, the dataset was divided by the seasons into batches. These were used in natural order from spring to winter, with each season being represented as one long drive of a train and considered as a one image sequence captured during one drive of the robot.

### 4.2. Stromovka

**Figure 5:**
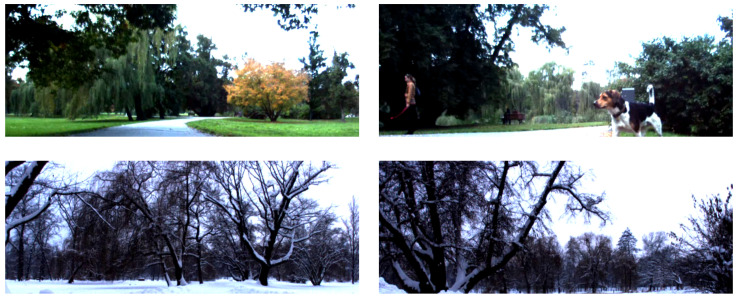
Images of different parts of the Stromovka dataset taken by the robot from the same place for each column.

For evaluation, we decided to use the Stromovka dataset [20], which exhibits significant seasonal changes in appearance as well as structures moving around. This dataset was recorded in Park Stromovka Prague, Czech Republic, using a P3-AT mobile robot equipped with Unibrain Fire-i601c camera [11]. Two drives were performed in 2011, one during summer the other during winter on a pathway of a publicly accessible suburban park show in Figure 5. The robot was driven manually, with each pass having a slightly different trajectory causing not only seasonal but also viewpoint changes. Use Stromovka dataset contains 500,1024 × 384 pixel image pairs, which allows us to test the deployment of our method on different resolutions and aspect ratios using the network trained on 512 × 288 pixel images of the Nordland. The ground truth containing the horizontal image displacements of each summer/winter image pair at different locations was obtained by hand annotation.

### 4.3. North Campus Long-Term Dataset—“Carlevaris”

**Figure 6:**
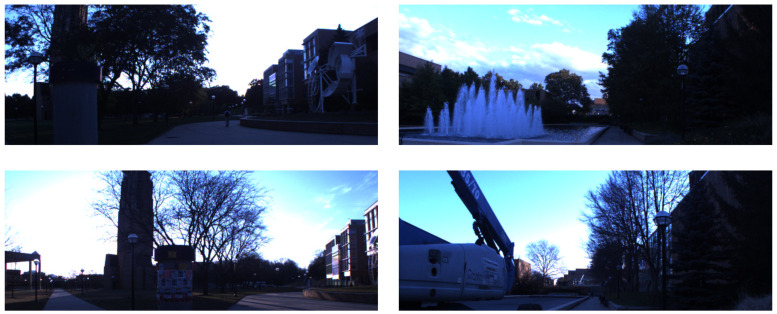
Examples of the Carlevaris dataset with seasonal variations (**left**) as well as structural changes (**right**).

The ‘North Campus Long-Term Dataset’ Was collected on the ground of the Michigan university using a robot-mounted omnidirectional camera, planar and 3D lidars and GPS-RTK for truth [53]. The whole dataset is comprised of 27 recording sessions that span over 15 months, capturing premises of the university campus outdoors and indoors following different paths at varying times and seasons. Two sequences were selected as in [20] spaced half a year apart during February and August of 2012 illustration of the data be seen in Figure 6. These sequences were processed and hand-annotated to obtain a dataset with a similar structure as the Stromovka one. The final dataset has 538,1024 × 384 pixel image pairs captured during two recording sessions.

## 5. Experimental Evaluation

The first step of the evaluation methodology is aimed at emulating the teach-and-repeat scenario using the Nordland dataset. During the first iteration, a robot is guided by an operator to record a sequence of images. For this purpose, we used the first ‘spring’ sequence of the Nordland dataset, which we designate as sequence $s_{0}$. Then, the second, ‘summer’ (or $s_{1}$) sequence of the Nordland was used to emulate the first autonomous repeat, where the neural network is untrained (we denote this network’s weights as $w_{0}$. In an untrained state, the neural network outputs a uniform image displacement probability, which does not guide the feature matching. The feature matching, therefore, works as described in [20], and it provides a training set which we denote as $T_{01}$ and image displacements which we denote as $d_{01}$. The training set is then used to train the neural network, which now contains the weights that we denote as $w_{1}$.

In the second autonomous traversal, which is emulated by re-running the sequence $s_{2}$ contained in the ‘Fall’ Nordland sequence using the sequence $s_{0}$ as a map, the feature matcher is already using the output of the neural network trained in the previous step, constructing the training set $T_{02}$, which is used to further train the neural network and an image displacement sequence $d_{02}$, which can be used to evaluate the quality of navigation. Moreover, the stored data can also be used to construct an additional training set $T_{12}$ using the sequence $s_{1}$ as a map and sequence $s_{2}$.

The subsequent runs are analogous. With each new sequence $s_{n}$, one can construct several additional training sets $T_{in}$, where i∈0…n−1, and use them to train the neural network. This means that the number of training sets increases quadratically with each autonomous repeat. In our case, we used four sequences of the Nordland dataset, which allowed us to emulate three autonomous runs, resulting in six training sets allowing us to train three generations of the neural networks with weights denoted as $w_{1}$, $w_{2}$ and $w_{3}$. Due to the filtering of the self annotated images by matched feature count, only about 80% of possible image pairs were used for training. The quality of the three autonomous repeats are assessed by comparing the calculated image displacements between the actual runs $d(01,w_{0})$, $d(02,w_{1})$ and $d(03,w_{2})$ and the ground truth provided by the dataset. These differences of the calculated displacements and the ground truth are denoted as $e(01,w_{0})$, $e(02,w_{1})$ and $e(03,w_{2})$, respectively. These represent errors in displacement calculation for each image pair used in the three subsequent autonomous repeats, i.e., each $e(ij,w_{k})$ is a sequence of 14,123 numbers, each corresponding to an error of displacement estimation of an image pair in the Nordland dataset season 0 and 1 with the neural network generation of *k*. For comparison, we also calculated the errors $e(02,w_{0})$ and $e(03,w_{0})$, which correspond to the situation where no learning is employed, and the traversals are performed purely by traditional feature matching.

### Computational and Storage Requirements

During navigation, the whole VTRL was able to run at 25 Hz on Intel NUC with i7 CPU and external GTX1050 GPU without heavy memory usage. This is sufficient for real-time control of most robotic platforms. The training was performed using an RTX3080 graphics card and AMD 3950X CPU, where each iteration of training took about 8 min for each training round, being about 35 ms per image pair at 28 HZ.

For the training, not only computational power is required but storage of all the images as well. The whole dataset used on Nordland has 7 GB without any compression and consists of several hours of recorded rides. Considering the robot would have driven one path per day, the data consumption would be less than 1 TB per year. Such volumes are manageable with off-the-shelf SSD drives, which also offer fast access for training the networks.

## 6. Results and Discussion

### 6.1. Evaluating the Teach, Repeat and Learn Scheme

**Figure 7:**
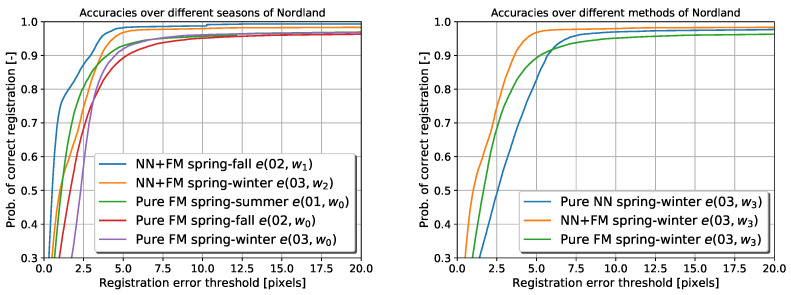
Error rates on the Nordland dataset using the visual teach and repeat with and without learning for each autonomous repeat (**left**). Error rates of the pure neural network output compared to pure feature matching and their combination (**right**).

The results are visualised in the same way as in [24,28,47]. Figure 7 displays the results by showing the probability of image registration error e, in pixels (px), being lower than a given threshold.

The left part of Figure 7 shows that the VTRL (NN + FM) scheme achieves higher accuracy of the image registration compared to the registration based on the feature matching alone. The right part of Figure 7 indicates that the image registration based on the combined neural network and feature matcher outperforms the standalone versions in terms of accuracy and robustness.

The comparison was conducted using a non-parametric Wilcoxon pair test, which does not pose any assumptions that might not be satisfied in our case. For two compared methods, the individual errors made by both methods were first paired over the locations where they appeared. The test was then conducted to reject the hypothesis that the methods performed equally well, which we did at a standard level of significance 0.05. Therefore we concluded that our results are significant. Because we were able to reject all the hypotheses using a non-parametric test, we did not study further whether a parametric one could be applied as additional testing of more assumptions would bring an unnecessary potential for Type II error.

### 6.2. Evaluating the Generalisation of the Trained Network

**Figure 8:**
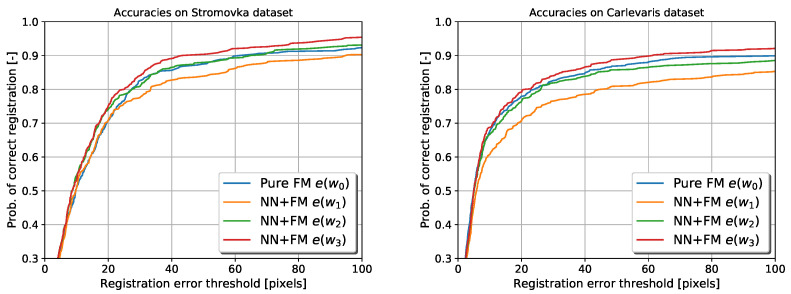
Probabilities of correct image registration when using the Nordland-trained network on Stromovka dataset (**left**) and Carlevaris dataset (**right**).

Although the results shown in Figure 7 demonstrate the performance of the VTRL in an environment the robotics operating in, it does not indicate how the trained network would generalise in different environments. To provide an insight into the generalisation ability of the network, we have used the networks $w_{1},w_{2}$ and $w_{3}$, trained on the Nordland dataset, to augment the feature matcher on the Stromovka and Carlevaris datasets. Figure 8 shows that each iteration of the teaching improves the registration accuracy. However, all three runs are required to outperform the original method, which was actually trained in the Stromovka park [20]. This shows that while it is possible to achieve better results of the proposed method on images from a different environment and of a different size, aspect ratio and camera, it requires the neural network to be trained by several runs.

### 6.3. Performance Comparison to Supervised Training

**Figure 9:**
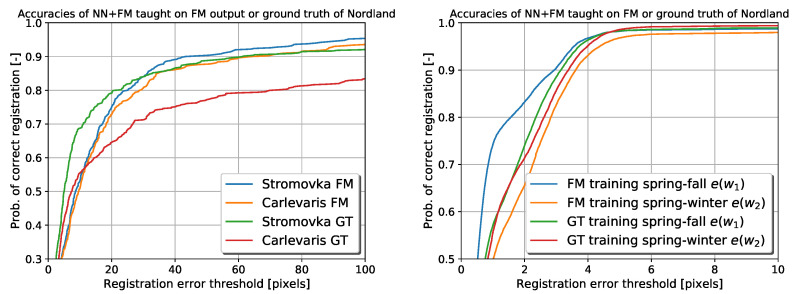
Probabilities of correct image registration on Nordland, Stromovka and Carlevaris datasets when using supervised and unsupervised training.

Finally, we compare the performance of the VTRL scheme to the case where the network is trained using hand-annotated data rather than the output of the feature matcher. The results shown in Figure 9 indicate that the proposed automatic training of the neural network yields better results on Carlevaris and Stromovka datasets than using the “ground truth” hand annotations. However, this is not present if the same experiment for testing is in the same environment, such as Nordland. We suspect that this is caused by the Nordland dataset being easier for humans to label, which has rails and watermarks, which are allowed through in this stage. Another explanation is that usage of filtering the data based on the feature count, which rejects potentially incorrect training samples, has a major impact on the training. An additional explanation is that the automatic feature annotation is not so precise, which allows for small annealing of the input data making it more robust to changes in the environment.

## 7. Conclusions

We propose a novel method for obtaining accurate image registration of two images captured from the same location in a changing environment, aimed at application for visual teach-and-repeat navigation of autonomous mobile robots. This method incorporates online self-supervised learning techniques to improve vision-only navigation in changing environments, combining neural network and feature matching methods. We show that our new method can autonomously learn using data from different seasons and improve its performance with every passage through the environment. We also show that the network trained in one environment can improve the performance of the visual teach-and-repeat scheme in other environments as well. Additionally, automatic annotation and filtering of the training data that was implemented yields better results than training on all data using ground truth. Moreover, we will improve the proper selection of images for training and using additional information such as output histogram from the FM should be explored. In closing, (i) the proposed visual-based navigation method, which combines feature matching methods and neural network, (ii) does not require hand annotation and learns automatically, (iii) and outperforms the model trained on ground truth. (iv) furthermore, the proposed method yields generality by learning data from other datasets and seasons.

## Data Availability

Datasets and codes of the work presented here are available at this link: https://github.com/rouceto1/VTRL (accessed on 1 March 2022).
